# Gunshot Wound of the Abdomen—Abdominal Section and Suturing Wounds of the Intestines

**Published:** 1890-08

**Authors:** A. S. Whittaker

**Affiliations:** Biltmore, N. C.


### GUNSHOT WOUND OF THE ABDOMEN—ABDOMINAL SECTION AND SUTURING WOUNDS OF THE INTESTINES. SUCCESSFUL.

BY A. S. WHITTAKER, M. D., BILTMORE, N. C.

I was called, on April 26, 1890, to see Charley Walker, aged 26, and found that he had received a gunshot wound, the ball entering one-fourth of an inch to the right of the umbilicus. He complained of a fluttering and smothering sensation about the heart, and began to feel sick at his stomach. There were pains in the hips and back, and very severe griping pains in the bowels. A sick headache and vomiting followed.

Upon examination I found that the ball had entered the abdomen, and all the symptoms indicated that the intestines had been wounded. I believed that the case demanded a surgical operation, and that no other means would save the man's life.

I called Dr. J. H. Williams, of Asheville, to assist me, and upon consultation he fully agreed with me as to the indications for operative interference. Chloroform was administered, and an incision on left of the umbilicus, extending a little above and below about three inches, made by Dr. Williams. The intestines were drawn out at the wounded portion, and it was found that two convolutions of the ileum, and the descending colon near the sigmoid flexure, had been injured making five distinct wounds. These were united with fine silk sutures, the threads including half an inch of the bowel tissue, and passing through the peritoneal and muscular coats. The intestines were put back, and the abdominal wound closed with silver wire sutures. Dressings of iodoform and borated cotton applied to the wound, and half grain of morphia administered. In two or three hours the patient was comfortable, and morphine was ordered every four hours to keep him quiet.

27th. Patient rested well last night, and after twenty-four hours was allowed a small amount of sweet milk.

28th. Doing well.

29th. Gave one and a-half drops of tincture of aconite every two hours. In the afternoon gave an enema of glycerine and water, and at stool the ball was passed.

For several days the temperature ranged from 98 to 101, and after May 3d was normal. In fifteen days the patient walked to Asheville, a distance of two miles, and soon said he felt as well as ever.

Being tired of the do-nothing plan, I determined in this case to explore the track of the ball and find it if possible, and if the intestines were injured, unite them. The result shows that this was the only course open to save his life. I am of the opinion that before long the prejudice against such operations will be successfully overcome, and the chances of the patients be largely enhanced in gunshot wounds of the abdomen.

Dr. Williams rendered me valuable assistance in the case, for which I am pleased to acknowledge my indebtedness.

